# Wideband, wide-angle coding phase gradient metasurfaces based on Pancharatnam-Berry phase

**DOI:** 10.1038/srep43543

**Published:** 2017-03-06

**Authors:** Qiqi Zheng, Yongfeng Li, Jieqiu Zhang, Hua Ma, Jiafu Wang, Yongqiang Pang, Yajuan Han, Sai Sui, Yang Shen, Hongya Chen, Shaobo Qu

**Affiliations:** 1 College of Science, Air Force Engineering University, Xi,an 710051, People’s Republic of China

## Abstract

A new concept of the coding phase gradient metasurface (CPGM) is proposed, which is constructed using the phase gradient metasurface as the coding elements. Different from the previous coding metasurface (CM), both the coding sequences and gradient phases in the coding elements are designed to manipulate the electromagnetic (EM) wave for the CPGMs, and thus the manipulation will be more flexible. As an example, wide-band, wide-angle CPGMs with zero and non-zero phase gradient based on Pancharatnam-Berry (PB) phase are achieved using the co-polarization reflection unit cells under circularly polarized (CP) wave incidence. Both theoretically calculated and numerically simulated scattering patterns of the designed CPGMs demonstrate the expected manipulations. Additionally, two kinds of random CPGMs with different phase gradients are designed for radar cross section (RCS) reduction, and the measured RCS reveals a good accordance with the simulation.

Over the past few years, the research of metamaterials have attracted much attention and achieved many applications, such as perfect lenses, invisible cloaks, imaging, perfect absorption and negative refraction^1^,^2^,^3^,^4^,^5^,^6^, but usually suffer from the bulky thickness and large loss. However, metasurfaces, a typical of two-dimensional metamaterials which usually constituted by inhomogeneous arrays of subwavelength resonators^7^, provide a promising candidate. Since Yu *et al*. proposed and achieved the phase gradient metasurfaces (PGMs) on the basis of the generalized versions of reflection and refraction law (Snell’s law)^6^, many applications in electromagnetic devices such as the optical vortexes, light bending, anomalous reflection and focusing^6^,^7^,^8^,^9^,^10^,^11^,^12^,^13^ have been achieved, showing a flexible manipulation ability of the EM waves.

In 2014, Cui *et al*. proposed the concept of the coding metasurface (CM), which can manipulate EM wave by binary coding elements^14^. It is of more freedom than the traditional metasurfaces and provides a new way to design the metasurface. CMs have been found comprehensive applications in microwave and terahertz frequencies^14^,^15^,^16^,^17^,^18^,^19^,^20^,^21^,^22^,^23^. Manipulation of polarization states, as well as broadband diffusion of terahertz waves have been achieved by anisotropic coding and multi-bit surfaces, respectively^15^,^16^,^17^,^18^. In microwave spectrum, based on digitally-controlled CM, one can achieve field-programmable beam reconfiguring^19^,^20^. So far, CMs have been studied widely, but only coding sequences of the phase are considered in the design of CMs.

In this work, we propose a concept of coding phase gradient metasurface (CPGM) with coding elements having a phase gradient. Compared with other CMs, the CPGM can manipulate EM wave by not only coding sequences but also the phase gradient of coding elements. To generate these CPGMs, we utilize the N-shaped element as the unit cell which has a co-polarization reflection characteristic under circularly polarized (CP) waves incidence^11^. Based on Pancharatnam-Berry (PB) phase, we then generate the coding elements for 1-, 2- and multi-bit CPGMs^22^,^23^. Two kinds of CPGMs with different phase gradients are considered. Both theoretic analysis and numerical simulation indicate that our CPGMs have a more flexible capability of manipulating the EM wave.

## Results

### Theoretical analysis

To illustrate the concept of the CPGM, we consider a general metasurface with N × N array of coding elements. And each element consists of M × M array of unit cells. Since the CM consists of equivalent homogeneous structures, it can be considered as a passive array antenna and the coding element as a sub-array antenna. When it is illuminated by normally incident EM wave, the far-field function can be expressed as:


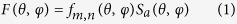


where *θ* and *φ* are the elevation and azimuth angles of the reflected wave. *f*_*m,n*_(*θ, φ*) is the primary pattern which expresses the vector properties of far-field like polarization and directional pattern. *S*_a_(*θ, φ*) is the array pattern which is a scalar quantity. As for the CPGM designed using co-polarization reflection unit cells under CP wave incidence, the array pattern *S*_a_(*θ, φ*) for CP wave incidence with incident elevation angle *θ*_*i*_ and azimuth angle *φ*_*i*_, the far-field function can be expressed as:


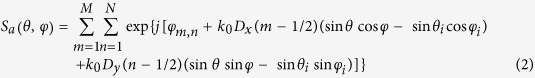


where *k*_*0*_ is the wave vector, *φ*_*m,n*_ is the reflection phase of each coding element, *D*_*x*_ and *D*_*y*_ are the size of the coding element in *x* direction and *y* direction, respectively^14^.

For traditional CMs, the coding elements usually consist of a M × M array of unit cells with the same reflectivity and reflection phase. Thus the primary pattern *f*_*m,n*_(*θ, φ*) is usually considered as a constant while calculating the far-field function. In other words, the manipulation of the far-field scattering pattern only depends on the array pattern *S*_*a*_(*θ, φ*). In fact, one can manipulate the EM wave with more freedom if the primary pattern is also designed. Based on such consideration, we propose the concept of the CPGM, in which both the primary pattern *f*_*m,n*_(*θ, φ*) and the array pattern *S*_*a*_(*θ, φ*) can be simultaneously designed. The modulation of the primary pattern *f*_*m,n*_(*θ, φ*) is realized by the PGM according to the generalized version of reflection law, and the modulation of the array pattern *S*_*a*_(*θ, φ*) by the coding sequences. The modulated primary pattern of the coding element can be written as *g*_*m,n*_(*θ, φ*). The main lobe direction (*θ*_*a*_, *φ*_*a*_) of primary pattern can be derived according to the generalized version of reflection law,


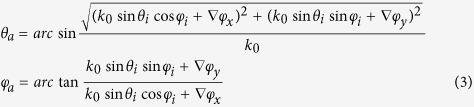


where *θ*_*a*_ and *φ*_*a*_ are elevation and azimuth angles of the main lobe for primary pattern, ∇*φ*_*x*_ = *dφ*_*x*_/*dx* and ∇*φ*_*y*_ = *dφ*_*y*_/*dy* are the phase gradient along *x* and *y* direction, respectively. The *dφ*_*x*_ and *dφ*_*y*_are the phase difference between adjacent unit cells in *x* direction and *y* direction, *dx* and *dy* are the length and width of unit cell, respectively.

**Figure 1:**
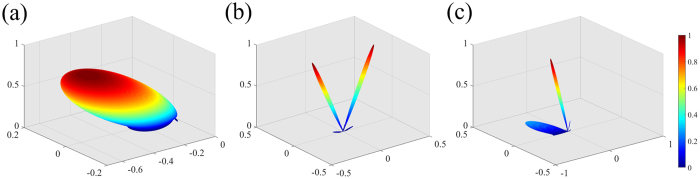
The 3-D far-field scattering patterns calculated by MATLAB. (**a**) The primary pattern of the coding element with one dimensional phase gradient ∇*φ*_*x*_. **(b)** The array pattern with 010101…/010101… coding sequence. **(c)** The final pattern *F(θ, φ*) of the CPGM.

To clearly illustrate the working mechanism of the proposed CPGM, we calculated the primary pattern *g*_*m,n*_(*θ, φ*) of the coding element with one dimensional phase gradient ∇*φ*_*x*_, the array pattern *S*_*a*_(*θ, φ*) with 010101…/010101… coding sequence and the final pattern *F(θ, φ*) of the CPGM using MATLAB as shown in Fig. 1. From the Fig. 1, we can find that the final pattern is resulted from the composition of the primary pattern and the array pattern, and the scattering pattern of the traditional CMs can be further manipulated by modulating the primary pattern of the consisted coding elements. In addition, the primary pattern of the coding elements can be designed to be a focusing beam, an out-plane anomalous reflected beam and other beams. In this work, we designed the CPGM using the coding element with one dimensional constant phase gradient ∇*φ*_*x*_. As the phase gradient ∇*φ*_*x*_ = 0, the CPGM will degenerate into the traditional CMs.

Furthermore, when the CPGM is illuminated by the CP wave, the reflected beam can be expressed as:


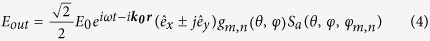


where “+” is for the left-handed circularly polarized (LCP) wave incidence, and “−” for the right-handed circularly polarized (RCP) wave incidence. Under the linearly polarized (LP) wave incidence, the reflected beam can be described as follows:


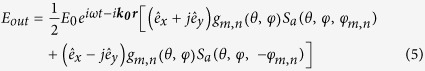


### Co-polarization reflection unit cell under CP wave incidence

**Figure 2:**
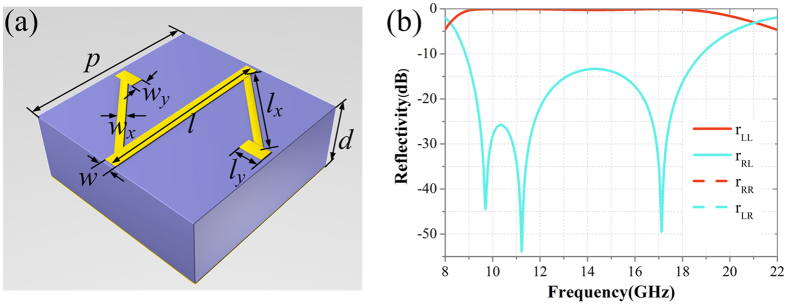
Design of the unit cell. (**a**) The perspective view of the unit cell co-polarization reflection unit cell under CP wave incidence. **(b**) The co-polarization and cross polarization reflectivity of the unit cell under CP wave normal incidence.

In order to achieve wideband, and wide-angle CPGM, the co-polarization reflection unit cell under the CP wave incidence (hereafter referred to as the unit cell) is employed to design the super unit of the PGM, which is used as the coding element of the CPGM. In this work, the unit cell (Fig. 2(a)) is composed of the N-shaped metallic pattern, dielectric substrate (*ε*_*r*_ = 2.65, tan *δ* = 0.001) and metallic ground. The repetition period of the unit cell is *p* = 5.2 mm. The thickness of the dielectric substrate is *d* = 3 mm. The thickness of both the metallic pattern and background is *t* = 0.017 mm. The geometrical parameters of the unit cell are *l* = 5 mm, *w* = 0.35 mm, *l*_*x*_ = 3.288 mm, *w*_*x*_ = 0.2 mm, *l*_*y*_ = 0.65 mm and *w*_*y*_ = 0.36 mm. The co-polarization reflectivity and cross polarization reflectivity under LCP and RCP waves normal incidence was calculated using Commercial software CST Microwave Studio. Figure 2(b) gives the simulated result in the frequency range of 8–22 GHz, where *r*_*LL*_/*r*_*RR*_ and *r*_*RL*_/*r*_*LR*_ represent the co- and cross polarization reflectivity under LCP and RCP waves normal incidence, respectively. The results demonstrate the high-efficiency, wide-band co-polarization reflection of the unit cell under CP wave incidence. In addition, the co-polarization reflectivity is independent of the incidence angle.

**Figure 3:**
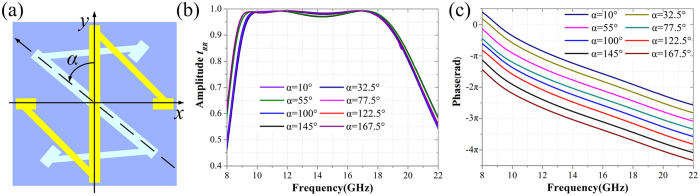
Co-polarization reflection manipulations of the unit cell. (**a**) Description of the rotation angle *α*. (**b,c**) The simulated amplitude and phase of co-polarization reflection coefficients of the unit cells with different rotating angle *α* under RCP wave normal incidence.

Based on the PB phase, the co-polarization reflection phase under the CP wave incidence can be manipulated with great freedom. In detail, the co-polarization reflection phase shift of the unit cell is Δ*φ* = ±2*α*, where “+” is for LCP wave, “−” is for RCP wave, and *α* is the rotation angle of the N-shaped metallic pattern (see in the inset of Fig. 3(a)). Figures 3(b,c) give the simulated amplitude and phase of the co-polarization reflection coefficient with different rotation angles under RCP wave incidence, respectively. Obviously, as the rotation angle *α* is changed from 10° to 167.5° with a 22.5° step width, the amplitudes of the co-polarization reflection coefficients are all larger than 0.97 in a wide frequency region 9.5–18 GHz, but the phases increase with a 45° step width in the whole frequency region. Besides, the co-polarization reflectivity and the co-polarization reflection phase shift are independent of the incidence angle.

### Wideband, wide-angle zero-gradient CPGMs

**Figure 4:**
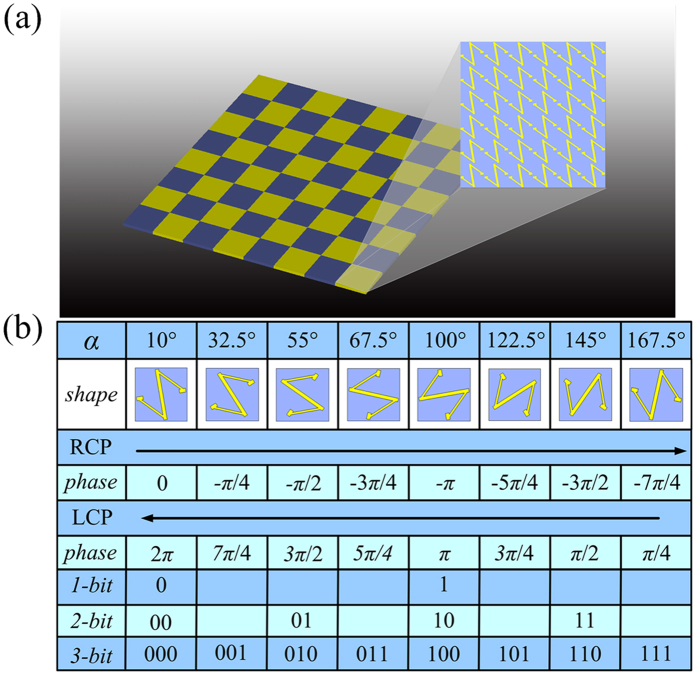
Detailed structure of unit cells and coding elements for the zero-gradient CPGM. (**a**) The makeup of the coding element with phase gradient ∇*φ*_*x*_ = 0. (**b**) The design of the coding elements for the 1-, 2-, 3-bit CPGMs.

We now turn to the design of the CPGMs utilizing the above N-shaped unit cell. The zero-gradient CPGM consisting of the coding elements with phase gradient ∇*φ*_*x*_ = 0 is firstly considered. A 6 × 6 array of equal phase unit cells is used as the coding element of the zero-gradient CPGM, as shown in Fig. 4(a). Based on PB phase, the co-polarization reflection phase under the CP wave incidence can be freely manipulated via rotating the N-shaped metallic pattern in-plane, and yet the co-polarization reflectivity almost keeps unchanged. Thus arbitrary bit coding elements can be easily built according to the co-polarization reflection phase. The design of the coding element with the phase gradient ∇*φ*_*x*_ = 0 is depicted in Fig. 4(b).

**Figure 5:**
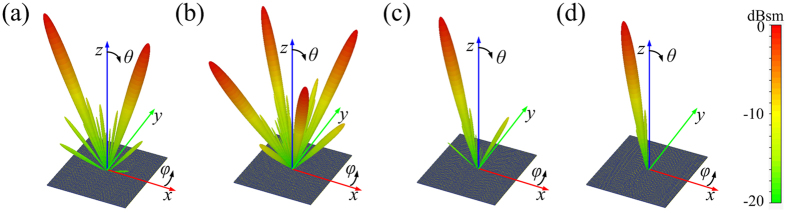
The 3D far-field scattering patterns of CPGMs with different coding sequences under RCP wave normal incidence. (**a**) The 3D far-field scattering patterns of 1-bit CPGM with 010101…/010101…coding sequence. (**b**) The 3D far-field scattering patterns of 1-bit CPGM with the chessboard distribution. (**c**) The 3D far-field scattering patterns of 2-bit CPGM with 00011011…/00011011… coding sequence. (**d**) The 3D far-field scattering patterns of 3-bit CPGM with coding elements distributed ordinal.

The zero-gradient CPGMs can reflect the incident CP wave into arbitrary directions by designing the coding sequences. To verify the characteristic of designed zero-gradient CPGM, numerical simulations were performed using CST Microwave Studio to calculate the bi-static radar cross section (RCS) of the 1-bit, 2-bit, and 3-bit CPGMs with designed coding sequences under RCP wave normal incidence at 15 GHz. The simulated 3D far-field scattering patterns are shown in Fig. 5. The 1-bit CPGM with 010101…/010101… coding sequence reflects the normal incident RCP wave into two symmetrically directions (Fig. 5(a)), and with chessboard distributed 010101…/101010… coding sequence reflects the normal incident RCP wave into four symmetrically directions as shown in Fig. 5(b). The 2-bit CPGM with the 00011011…/00011011… coding sequence anomalously reflects the normal incident RCP wave into one direction as shown in Fig. 5(c). In Fig. 5(d), the 3-bit CPGM with coding elements ordinal distribution anomalously reflects the normal incident RCP wave into one direction with a smaller reflection angle compared with the 2-bit CPGM. All of these suggest that the zero-gradient CPGMs constituted by the co-polarization reflection unit cells can manipulate EM wave by coding sequences.

**Figure 6:**
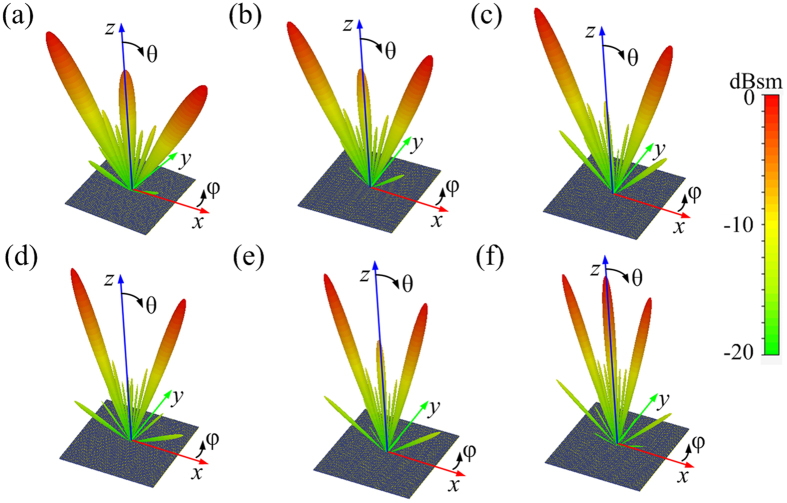
The 3D far-field scattering patterns of the 1-bit CPGM with 010101…/010101…coding sequence under RCP wave normal incidence at different frequency. (**a–f**) The far-field scattering patterns of the 1-bit CPGM with 010101…/010101…coding sequence at 10, 12, 14, 16, 18 and 20 GHz.

Due to the non-dispersive phase displacement of the unit cell, the CPGMs have a wide operation band-width. To verify this wideband characteristic, we performed numerical simulations for the bi-static RCS of the 1-bit CPGM with 010101…/010101… coding sequence at different frequencies (10, 12, 14, 16, 18 and 20 GHz) under RCP wave normal incidence, as shown in Fig. 6. Obviously, it is indicated that at all these six frequencies, the normal incidence RCP waves are reflected into two symmetrically directions. Since the co-polarization reflection phase difference between two unit cells and the co-polarization reflectivity of the unit cells are not perfect near the frequencies *f* = 10 GHz and 20 GHz, the anomalous reflection efficiency of the CPGM near the two frequencies is reduced and the specular reflection is increased.

**Figure 7:**
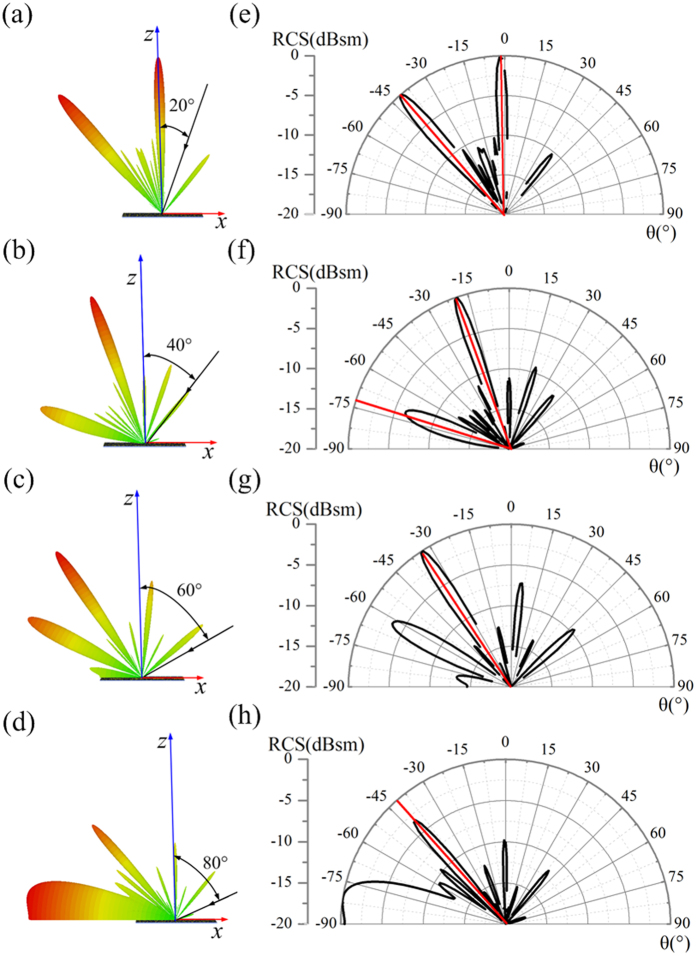
The 3D far-field scattering patterns and polar scattering patterns of the 1-bit CPGM with 010101…/010101… coding sequence under RCP wave incidence with different incidence angle. (**a–d**) The 3D far-field scattering patterns of the 1-bit CPGM with different incidence angles (*θ* = 20°, 40°, 60°, 80°) at 15 GHz. (**e–h**) The polar scattering patterns of the 1-bit CPGM with different incidence angles (*θ* = 20°, 40°, 60°, 80°) at 15 GHz.

In addition, as for the traditional CMs, the anomalous reflection is always incidence-angle dependent mainly because of the incidence-angle dependent phase difference between the coding elements. But the co-polarization reflection phase difference achieved based on PB phase between the designed unit cells is independent with the incident angle. Therefore, the designed CPGMs are independent with the incidence angle. The bi-static RCS of 1-bit CPGM with 010101…/010101… coding sequence under RCP wave incidence with different incidence angles (*θ*_*i*_ = 20°, 40°, 60° and 80°) at 15 GHz is simulated and shown in Fig. 7, where the black arrow lines describe the incident directions. It can be found that the manipulation of EM waves is valid under the 60°-angle incidence, displaying a wide-angle feature.

### Non-zero-gradient CPGMs

**Figure 8:**
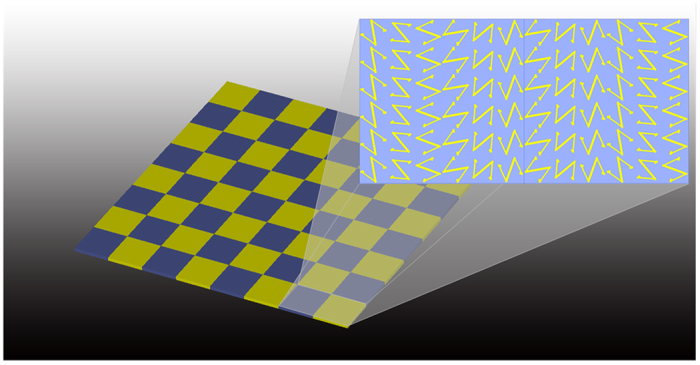
The detailed structure of the coding element with non-zero phase gradient.

As for the above zero-gradient CPGMs, the manipulation of reflected wave only relies on the coding sequences. In this section, we discussed the CPGMs consisting of the coding element with one dimensional phase gradient ∇*φ*_x_ = (π/3)/*dx*. In this case, the manipulation of the reflected wave relies on both the phase gradient of the unit cells and the coding sequences. In other words, the reflected wave can be manipulated via modulating the primary pattern *g*_*m,n*_(*θ, φ*) and the array pattern *S*_*a*_(*θ, φ*). As an example, Fig. 8 gives a detailed view of the 1-bit coding elements. Each coding element consists of 6 × 6 unit cells with a phase gradient in the *x* direction, and thus the phase difference between two adjacent unit cells is *dφ*_*x*_ = *π*/3. The phase gradient is achieved via manipulating the co-polarization reflection phase under the CP wave incidence based on PB phase.

For the 1-bit CPGM, if we defined the blue area in Fig. 8 as the coding element “0”, the “1” can be obtained by rotating the N-shaped unit cell with *α* = 90°, as shown in the inset of Fig. 8. Hence the coding elements “0” and “1” have opposite co-polarization reflection phase, but the phase gradient can be kept all the same. For the 2-bit CPGM, the “00” coding element is the “0” coding element of the 1-bit CPGM, and the “01”, “10” and “11” coding elements are obtained by rotating the unit cells of the “00” element with *α* = 45°, *α* = 90° and *α* = 135°. Hence the coding elements “00”, “01”, “10” and “11” have four different co-polarization reflection phase 0, −*π*/2, −*π* and −3*π*/2 with the identical phase gradient. Similarly, for the multi-bit CPGM, the coding elements can be designed in the same way.

**Figure 9:**
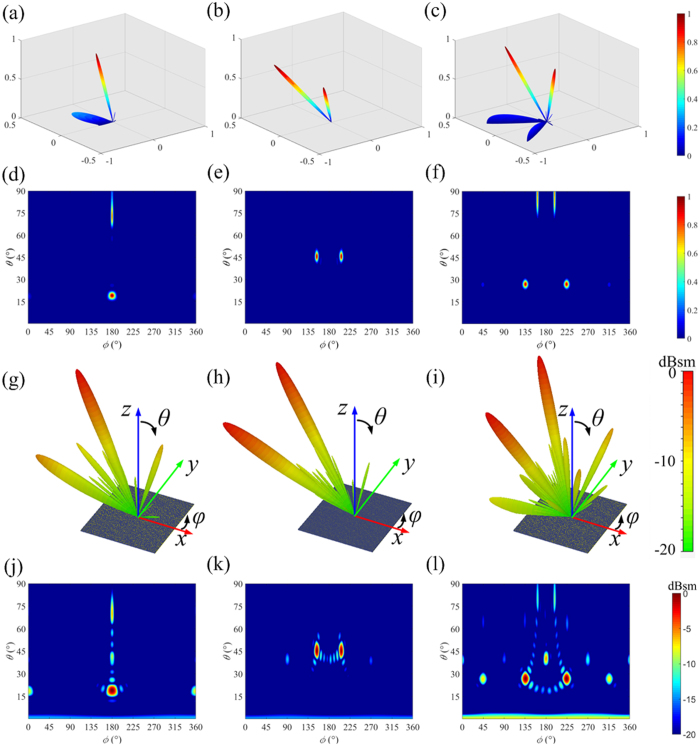
The far-field scattering patterns of 1-bit CPGMs with different coding sequence under RCP wave normal incidence. (**a–c**) The 3D far-field scattering patterns of 1-bit CPGMs with 010101…/010101…coding sequence; 000000…/111111…coding sequence and the chessboard distributed coding sequence calculated by MATLAB. (**d–f**) The 2D far-field scattering patterns of the 1-bit CPGMs with 010101…/010101…coding sequence; 000000…/111111…coding sequence and the chessboard distributed coding sequence calculated by MATLAB. (**g–i**) The 3D far-field scattering patterns of the 1-bit CPGMs with 010101…/010101…coding sequence, 000000…/111111…coding sequence and the chessboard distributed coding sequence simulated by CST Microwave Studio. (**j–l**) The 2D far-field scattering patterns of the 1-bit CPGMs with 010101…/010101…coding sequence; 000000…/111111…coding sequence and the chessboard distributed coding sequence calculated by CST Microwave Studio.

The scattering patterns of the CPGMs were theoretically calculated using MATLAB and simulated by the CST Microwave Studio, respectively. The results for the 1-bit CPGMs under the RCP wave normal incidence at 15 GHz are shown in Fig. 9, where the results for the CPGM with 010101…/010101… coding sequence is given in Fig. 9(a) and (j). The “0” and “1” coding elements are periodically distributed along *x* direction. It is found that the CPGM reflected the normal incidence RCP wave into two directions symmetric about the main lobe direction of the primary pattern *g*_*m,n*_(*θ, φ*). The simulated far-field scattering pattern keeps in good accordance with the theoretically calculated results. Figures 9(b) and (k) give the results of 000000…/111111… coding sequence. The “0” and “1” coding elements are periodically distributed along *y* direction. The normal incidence RCP wave is efficiently reflected along two main directions symmetric about the *xoz* plane. Figures 9(c) and (l) show the results of the 1-bit CPGM with chessboard distribution of the “0” and “1” coding elements. It is observed that the normal incident RCP wave is reflected into four directions symmetric about the main lobe direction of the primary pattern *g*_*m,n*_(*θ, φ*).

**Figure 10:**
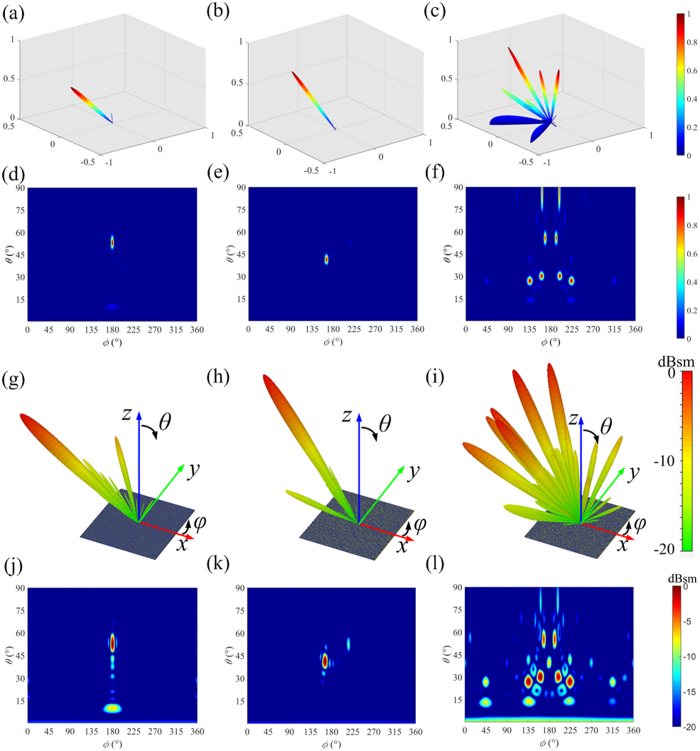
The far-field scattering patterns of 2-bit CPGMs with different coding sequence under RCP wave normal incidence. (**a–c**) The 3D far-field scattering patterns for the 2-bit CPGMs with 00011011…/00011011…coding sequence, 00…/01…/10…/11…coding sequence and the chessboard distributed coding sequence calculated by MATLAB. (**d–f**) The 2D far-field scattering patterns for the 2-bit CPGMs with 00011011…/00011011…coding sequence, 00…/01…/10…/11…coding sequence and the chessboard distributed coding sequence calculated by MATLAB. **(g–i)** The simulated 3D far-field scattering patterns for the 2-bit CPGMs with 00011011…/00011011…coding sequence, 00…/01…/10…/11…coding sequence and the chessboard distributed coding sequence. **(j-l**) The simulated 2D far-field scattering patterns for the 2-bit CPGMs with 00011011…/00011011…coding sequence, 00…/01…/10…/11…coding sequence and the chessboard distributed coding sequence.

As for the 2-bit CPGMs with different coding sequences, Fig. 10 gives the theoretically calculated scattering patterns by MATLAB and the bi-static RCS simulated using CST Microwave Studio under RCP wave normal incidence at the frequency *f* = 15 GHz. Figure 10(a) and (j) show the results for the 00011011…/00011011… coding sequence with the “00”, “01”, “10” and “11” coding elements periodically distributed along *x* direction, Fig. 10(b) and (k) for the 00…/01…/10…/11… coding sequence with the “00”, “01”, “10” and “11” coding elements periodically distributed along y direction, and Fig. 10(c) and (l) for the chessboard distribution with 00100111…/10001101…/01110010…./11011000… coding sequence. One can find that all the simulated results are in good accordance with the theoretically calculated results. In detail, the 00011011…/00011011… coding sequence reflects the normal incident RCP wave into one direction in *xoz* plane, the 00…/01…/10…/11… coding sequence reflects the normal incident RCP wave into one direction deviating from the *xoz* plane, and the chessboard distribution with coding sequence reflects the normal incident RCP wave into eight main directions. All the deflection directions can be easily derived from the calculated and simulated 2D scattering patterns given in Fig. 10(d–f) and (j–l).

**Figure 11:**
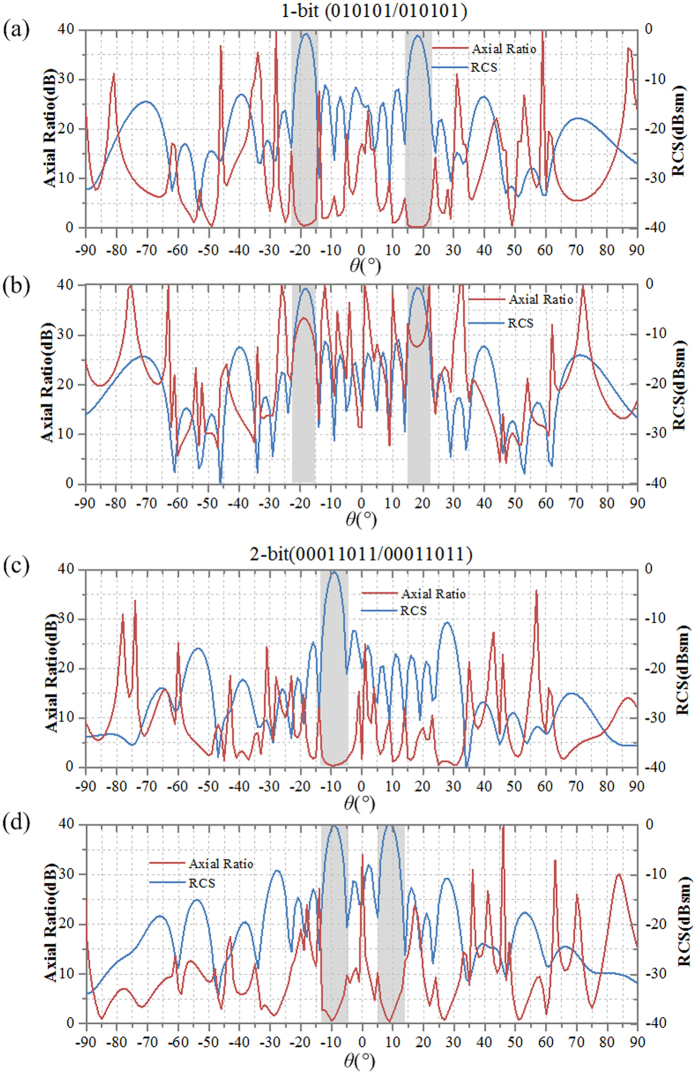
The axial ratio and scattering pattern of 1-bit CPGM with 010101…/010101… coding sequence and 2-bit CPGM with 00011011…/00011011… coding sequence under CP wave and LP wave normal incidence, respectively. (**a**) The axial ratio and scattering pattern of 1-bit CPGM with 010101…/010101… coding sequence under CP wave normal incidence. (**b**) The axial ratio and scattering pattern of 1-bit CPGM with 010101…/010101… coding sequence under LP wave normal incidence. (**c**) The axial ratio and scattering pattern of 2-bit CPGM with 00011011…/00011011… coding sequence under CP wave normal incidence. (**d**) The axial ratio and scattering pattern of the 2-bit CPGM with 00011011…/00011011… coding sequence under LP wave normal incidence.

The CPGMs above are designed under CP wave incidence. As the CPGMs are illuminated by LP waves, decomposing the LP incidence wave into two beams of CP waves (one beam of LCP wave and one beam of RCP), the polarization of the reflected waves can be derived according to the equations (4) and (5). If the reflected beams are symmetric under CP wave incidence, the mainly reflected beams will be LP wave under LP wave incidence. And that the mainly reflected beams will be converted into CP waves under LP incidence if the reflected beams are asymmetric under CP wave incidence. To demonstrate this conclusion, we simulated the axial ratio versus reflection angle of the reflected waves together with the far-field scattering pattern. The simulated axial ratio and scattering pattern of 1-bit CPGM with 010101…/010101… coding sequence and 2-bit CPGM with 00011011…/00011011… coding sequence under CP wave and LP wave normal incidence at 15 GHz are shown in the Fig. 11, where Fig. 11(a–c) give the simulated results under CP wave incidence, and Fig. 11(b–d) under LP wave incidence. In Fig. 11(a), the axial ratio of the two mainly reflected beams is nearly 0 dB. Thus the reflected beams are CP waves. In Fig. 11(b), The reflected waves are LP, because the axial ratio of the two mainly reflected beams is more than 28 dB. However, for the 2-bit CPGM with 00011011…/00011011… coding sequence, the reflected waves are all circularly polarized under CP and LP waves incidence, as shown in Fig. 11(b–d).

### Random CPGMs

By the CPGMs with random coding sequence, the incident wave can be reflected to all directions. For comparison, two kinds of 2-bit CPGMs with random coding sequence are designed for RCS reduction. The random coding sequence of the CPGMs composed of a 8 × 8 array of coding elements produced by MATLAB is given as follows,


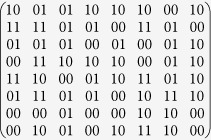


**Figure 12:**
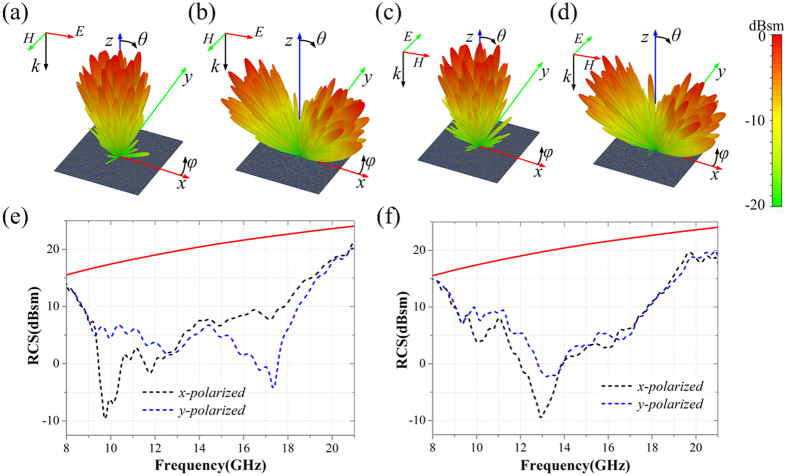
The 3D far-field scattering patterns and mono-static RCS of the two CPGMs under LP wave normal incidence. **(a,b**) The 3D far-field scattering patterns of two CPGMs under *x*-polarized wave incidence. **(c,d**) The 3D far-field scattering patterns of two CPGMs under *y*-polarized wave incidence. **(e)** Mono-static RCS of the zero-gradient CPGM under *x*-polarized and *y*-polarized wave normal incidence. **(f)** Mono-static RCS of the non-zero-gradient CPGM under *x*-polarized and *y*-polarized wave normal incidence.

According to this random coding sequence, the zero-gradient and non-zero-gradient CPGMs are designed, respectively. The simulated bi-static RCS of the two CPGMs for LP wave normal incidence at 15 GHz are shown in the Fig. 12, where (a) and (c) present the results for the zero-gradient CPGM, (b) and (d) present the results for non-zero-gradient CPGM. Obviously, the zero-gradient CPGM reflects the incident LP wave into multi-directions centre on the normal direction. And the CPGM with non-zero phase gradient reflects the normal incident LP wave into multi-directions centre on two symmetric main lobe direction of primary pattern *g*_*m,n*_(*θ, φ*). Accordingly, the mono-static RCS of the two CPGMs in the normal direction is highly reduced.

To validate the reduction efficiency of RCS, the mono-static RCS versus frequency range from 8 GHz to 21 GHz of the two CPGMs under LP wave normal incidence are calculated as given in Fig. 12(e,f). The red solid line is the theoretical mono-static RCS of the metallic plate with same size of the CPGMs calculated by formula: RCS = 10 × log_10_(4*πL*^4^/*λ*^2^), where *L* is the length of the square metallic plate and the *λ* is the wavelength. Compared with metallic plate, the RCS is reduced more than 10 dB at 9.72–18.53 GHz for the zero-gradient CPGM and 9.71–18.52 GHz for the non-zero-gradient CPGM under *x*-polarized wave normal incidence. And under *y*-polarized wave normal incidence, the RCS is reduced more than 10 dB at 9.10–19.02 GHz for the zero-gradient CPGM and 11.33–18.65 GHz for the non-zero-gradient CPGM.

## Experimental Verification

**Figure 13:**
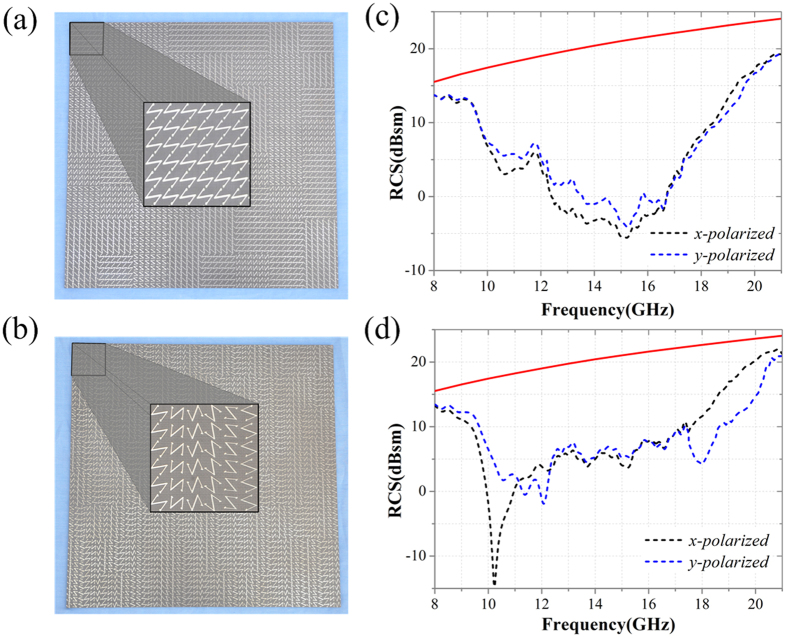
Photographs of the fabricated CPGM samples and the measured results. (**a**) Photographs of zero-gradient CPGM with the consisted coding elements in the inset. (**b**) Photographs of non-zero-gradient CPGM with the consisted coding elements in the inset. (**c**) Measured mono-static RCS versus frequency of the zero-gradient CPGM under LP wave normal incidence. (**d**) Measured mono-static RCS versus frequency of the non-zero-gradient CPGM under LP wave normal incidence.

To further verify the performances of the proposed CPGMs, two CPGMs with random coding sequence designed above are fabricated using the printing circuit board (PCB) technique. The sample photographs are shown in Fig. 13, where Fig. 13(a) shows the sample photograph of the zero-gradient CPGM, in which the inset gives a zoom view of the coding element, and Fig. 13(b) shows the sample photograph of the non-zero-gradient CPGM, in which the inset gives a zoom view of the coding element. The sizes of the two CPGMs are 249.6 × 249.6 mm^2^ composed of a 8 × 8 array of coding elements. The mono-static RCS versus frequency of the CPGM samples and the same sized metallic plate are measured as given in Fig. 13.

Mono-static RCS versus frequency of two CPGMs was measured to validate the efficiency of RCS reduction. Figures 13(c) show the measured mono-static RCS of two CPGMs under *x*-polarized and *y*-polarized wave normal incidence. The red solid line is the mono-static RCS of metallic plate with the same size of the CPGMs. Compared with the mono-static RCS of metallic plate, the reduction of RCS is more than 10 dB at 9.70–18.12 GHz for the zero-gradient CPGM and 9.83–19.12 GHz for the non-zero-gradient CPGM under *x*-polarized wave normal incidence. And under *y*-polarized wave normal incidence, the reduction of RCS is more than 10 dB at 9.92–19.84 GHz for the zero-gradient CPGM and 9.85–19.37 GHz for the non-zero-gradient CPGM. We can find that the measured mono-static RCS of the two CPGMs reveal a good accordance with the simulated result except for some small difference due to the measurement and fabrication errors. Both simulated and measured results demonstrate the great performance of the two CPGMs in RCS reduction.

## Conclusions

In summary, a novel concept of CPGM is proposed and demonstrated by the simulations and experiments. The CPGMs manipulate the reflected waves via modulating the array pattern *S*_a_(*θ, φ*) or the primary pattern *g*_*m,n*_(*θ, φ*). Accordingly, it is more flexible to manipulating the reflected waves. To validate the proposed CPGMs, the zero-gradient CPGMs and the non-zero-gradient CPGMs were designed. The unit cells of the co-polarization reflection metasurface under CP wave incidence were employed to design the CPGM. The co-polarization reflection phase manipulation of the unit cells is achieved based on PB phase. The simulated results of the zero-gradient CPGMs demonstrated the wide-band and wide-incidence-angle characteristic of the CPGMs based on PB phase. The simulated results of the non-zero-gradient CPGMs indicate that manipulations of the reflected waves depend on both the phase gradient of the coding element and the coding sequence. Finally, two 2-bit zero-gradient and non-zero-gradient CPGMs with identical random coding sequence were fabricated and measured. Both the simulated and measured results demonstrated the ability of the wide-band RCS reduction. The CPGMs provide a more flexible way for the manipulation of reflected waves.

## Methods

### Simulations

Electromagnetic simulations are performed using a commercially available software package, CST Microwave Studio. The bi-static RCS and mono-static RCS are calculated using the Integral equation solver with open boundary conditions along the *x, y* and *z* directions. The reflectivity and reflection phases of unit cells are simulated using the Frequency domain solver. In the simulations, unit cell boundary conditions in the *x* and *y* directions are used, and open (add space) boundary conditions in the *z* direction.

### Fabrication

The CPGMs are fabricated using the PCB photolithography. The commercial F4B dielectric substrates are employed as the dielectric layers and the 17-μm-thick copper films as the metal parts.

### Measurements

The measure of the RCS is carried in an anechoic chamber. The background noise of the anechoic chamber is under −60 dBsm. The CPGM sample is placed on a foam tower and is fixed uprightly into a foam base. The incident angle is changed when the foam tower is circumrotating. The mono-static RCS versus frequency for *x*- and *y*-polarized waves normal incidence is derived from the measured results.

## Additional Information

**How to cite this article:** Zheng, Q. *et al*. Wideband, wide-angle coding phase gradient metasurfaces based on Pancharatnam-Berry phase. *Sci. Rep.* **7**, 43543; doi: 10.1038/srep43543 (2017).

**Publisher's note:** Springer Nature remains neutral with regard to jurisdictional claims in published maps and institutional affiliations.
